# Integrative methylome and transcriptome analysis reveals epigenetic regulation of Fusobacterium nucleatum in laryngeal cancer

**DOI:** 10.1099/mgen.0.001221

**Published:** 2024-03-27

**Authors:** Xiaohui Yuan, Hui-Ching Lau, Huiying Huang, Chi-Yao Hsueh, Hongli Gong, Liang Zhou

**Affiliations:** 1 Department of Otorhinolaryngology Head and Neck Surgery, Eye and ENT Hospital, Fudan University, Shanghai, PR China; 2 Shanghai Key Clinical Disciplines of Otorhinolaryngology, Shanghai, PR China

**Keywords:** functional enrichment analysis, *Fusobacterium nucleatum*, laryngeal cancer, methylome, transcriptome

## Abstract

The aetiological mechanisms of *Fusobacterium nucleatum* in laryngeal cancer remain unclear. This study aimed to reveal the epigenetic signature induced by *F. nucleatum* in laryngeal squamous cell carcinoma (LSCC). Combined analysis of methylome and transcriptome data was performed to address the functional role of *F. nucleatum* in laryngeal cancer. Twenty-nine differentially expressed methylation-driven genes were identified by mapping the methylation levels of significant differential methylation sites to the expression levels of related genes. The combined analysis revealed that *F. nucleatum* promoted Janus kinase 3 (JAK3) gene expression in LSCC. Further validation found decreased methylation and elevated expression of JAK3 in the *F. nucleatum-*treated LSCC cell group; *F. nucleatum* abundance and JAK3 gene expression had a positive correlation in tumour tissues. This analysis provides a novel understanding of the impact of *F. nucleatum* in the methylome and transcriptome of laryngeal cancer. Identification of these epigenetic regulatory mechanisms opens up new avenues for mechanistic studies to explore novel therapeutic strategies.

**Impact Statement**

Laryngeal squamous cell carcinoma (LSCC) with metastasis has a poor prognosis. Our previous study revealed that *Fusobacterium nucleatum* promoted LSCC progression and metastasis by suppressing TGFβR2 expression. It is of note that restoring TGFβR2 expression caused an inexplicable multisite metastasis, proving that *F. nucleatum* promotes tumour metastasis via other routes. Here, we attempted to identify a new mechanism responsible for *F. nucleatum*-mediated metastasis. Combined analysis of the DNA methylome and transcriptome was used to understand the aetiological mechanisms of *F. nucleatum* in laryngeal cancer. We found that *F. nucleatum* treatment led to altered DNA methylation patterns in laryngeal cancer, and hence affected gene expression. This study is the first to examine the *F. nucleatum*-mediated methylation profile of laryngeal cancer. We identified the whole genome-wide methylation map and screened differentially expressed genes. This analysis provides a novel understanding that *F. nucleatum* plays a role as an epigenetic regulater in the metastasis of LSCC. The identification of these methylation patterns offers new possibilities for mechanistic studies to explore therapeutic strategies in laryngeal cancer.

## Data Summary

The datasets generated during the current study are available in the NCBI GEO database (https://www.ncbi.nlm.nih.gov/geo/) under accession number GSE236237.

## Introduction

Laryngeal squamous cell carcinoma (LSCC) is a prevalent type of head and neck cancers, responsible for 100 000 deaths every year [1]. Laryngeal cancer seriously affects patients’ survival quality, including vocalization and breathing, because of the larynx’s unique anatomical structure and function and has become a serious public health issue [2]. Non-surgical treatment modalities, such as concurrent chemoradiation and induction chemotherapy plus radiotherapy, have quickly emerged for preserving laryngeal function. Despite research achievements in therapy over the decades, the prognosis has not significantly improved for patients with advanced or metastasized disease [3]. Tumour metastasis remains an obstacle in cancer therapy and correlates with poor prognosis in patients with LSCC. Determining the precise molecular mechanism underlying tumour metastasis is necessary for progress in therapeutic interventions to inhibit tumour metastasis, which will improve the prognosis of patients with LSCC.

Host–microbiome interactions have attracted much attention in modulating health and disease. In recent decades, the microbiota has been recognized as an essential contributing factor in the initiation and progression of cancers. *Fusobacterium nucleatum *has been demonstrated to be associated with numerous tumour types [4,6]. The mechanisms of *F. nucleatum* involvement in colorectal metastasis have been elucidated. Specifically, *F. nucleatum* promotes endothelial adhesion and extravasation of tumour cells [7] and contributes to expression of tumour-derived CCL20, which is conducive to M2 macrophage infiltration [8], resulting in metastasis of colorectal cancer (CRC). Our previous study reported a higher abundance of *F. nucleatum* in LSCC tissues than in normal tissues, which confirmed the relationship between *F. nucleatum* and laryngeal tumorigenesis [9]. However, it is still unclear exactly how *F. nucleatum* has a pro-metastatic impact on laryngeal cancer at the molecular level.

Cancer development results from an accumulation of genetic and epigenetic abnormalities. Studies have shown that DNA methylation modulates gene transcription and acts as the key element in epigenetic modifications. The regulation of cellular functions and tumorigenesis is heavily dependent on aberrant DNA methylation [10,12]. Microbiota may exert a direct or indirect effect on the epigenome. Bacteria can modify host methyl-donor levels by interfering with choline metabolism, affecting DNA methylation patterns [13]. Recently, *F. nucleatum* was reported to promote CRC metastasis by reducing m^6^a modifications [14]. There is an imperative need to explore the regulation of *F. nucleatum* in the epigenetic inheritance of laryngeal cancer and whether it enhances tumour metastasis via specific mechanisms in LSCC.

In this study, we aimed to investigate the features by which *F. nucleatum* regulates DNA methylation, thus regulating gene expression. A combined analysis of the DNA methylome and transcriptome was conducted to further understand the aetiological mechanisms of *F. nucleatum* in laryngeal cancer.

## Methods

### Cell culture and bacterial strain

The AMC-HN-8 human laryngeal carcinoma cell line was a kind gift from Professor S. Y. Kim of Samsung Medical Center, Korea [15]. AMC-HN-8 cells were cultured using complete RPMI-1640 medium (HyClone) supplemented with 1 % penicillin-streptomycin, 10 % FBS and mycoplasma prevention reagent (Yeasen). The culture conditions were 37 °C with 5 % CO_2_. *F. nucleatum* strain ATCC 25586 was purchased from the American Type Culture Collection (ATCC). Before being cultured with cells, *F. nucleatum* was grown in thioglycollate medium (without agar) under anaerobic conditions at 37 °C for 72 h. Two groups were used: the control group (Ctrl) and the *F. nucleatum* group; cells in the *F. nucleatum* group were treated with the *F. nucleatum* strain (m.o.i.=500 : 1). AMC-HN-8 cells were seeded in a six-well plate at a density of 1×10^6^ ml^−1^ and cultured at 37 °C with 5 % CO_2_. After 24 h, AMC-HN-8 cells were treated with *F. nucleatum* (5×10^8^ c.f.u. ml^−1^, m.o.i.=500 : 1) and cultured at 37 °C with 5 % CO_2_ for an additional 24 h. Next, the cells were subjected to DNA and RNA extraction. Three biological replicates, each with three technical replications, were set per group.

### DNA methylome analysis

After extraction of total DNA from the AMC-HN-8 cell line using a QIAamp DNA Mini Kit (Qiagen), DNA methylation analysis was conducted with the Illumina Infinium MethylationEPIC (850K) BeadChip (Illumina). The ChAMP (version 2.12.4) package in R was used to obtain raw data. Next, the raw data were normalized with the Beta Mixture Quantile (BMIQ) method. Statistical differences in continuous variables between two groups were compared by a t-test. Significantly differential methylation sites (DMSs) were identified by a threshold of |delta beta|>0.1 and *P*<0.05. The gene region harbouring the probe was divided into an intergenic region (IGR), transcription start site (TSS) 1500, TSS200, 5′ untranslated region (UTR), first exon (1stExon), body, exon boundaries (ExonBnd) and 3′UTR. The CpG island region included N shelf, N shore, CpG island, S shore and S shelf. N and S shores are defined as regions 2 kb up- and downstream from the CpG island, while N shelf and S shelf are 2–4 kb from the CpG island. Open sea denotes isolated genomic regions lacking specific designation.

### Transcriptome analysis

Total RNA from AMC-HN-8 cell lines was extracted using the AG RNAex Pro Reagent (Accurate Biotechnology) and transcriptome sequencing was conducted on the Illumina HiSeq X Ten platform (Illumina). Library preparation and bioinformatic analysis were conducted on the Illumina HiSeq X Ten platform (Illumin). Raw reads were processed using Trimmomatic to obtain clean reads. The clean reads were mapped to the reference genome using hisat2. The fragments per kilobase of exon model per Million mapped fragments (FPKM) value of each gene was calculated using cufflinks. The functions estimateSizeFactors and nbinomTest were used with the DESeq (2012) R package. Significant differentially expressed genes (DEGs) were idenfitied by *P*<0.05 and |log_2_ fold change|>1.

### Integrative analysis of methylome and transcriptome analysis

In the integrated methylome and transcriptome analysis, methylation-driven genes (MDGs) were identified by correlating the gene name of the DEGs with the DMS results based on the criterion of *P*<0.05, |delta beta|>0.05, and fold change >1.5 or <0.5.

### Functional enrichment analysis

Function and biological pathways of DEGs and MDGs were clarified using Gene Ontology (GO) [16] and the Kyoto Encyclopedia of Genes and Genomes (KEGG) [17]. After DEGs were identified, GO and KEGG enrichment analyses were conducted to describe gene functions. The number of genes included in each functional item was counted and the significance of gene enrichment in each functional item was calculated based on a hypergeometric distribution. The interaction network was obtained using the STRING database to analyse the interaction relationship between DEGs. To screen for key genes, KEGG functional enrichment and methylation data were mapped to the interaction network. Cytoscape 3.9.1 software was used for visualization of the interaction network.

### Bisulfite sequencing PCR (BSP)

DNA was bisulfite-converted using the EpiTect Fast DNA Bisulfite Kit (Qiagen). The primer was designed to detect the targeted gene. Then, DNA that had been bisulfite-converted was amplified using PCR. The following primers were used: Janus kinase 3 (JAK3)-F, 5ʹ-GAATGAGAGTTTGTGTGTGTTT-3ʹ, JAK3-R, 5ʹ-AACTCAAACCCTAAATCAAATC-3ʹ. The PCR products were cloned into the pMD18-T vector. Then, from each group, ten clones were randomly selected and sequenced. Three technical replicates were performed in each group.

### Clinical samples

Fresh tumour tissues from 30 patients with LSCC who underwent laryngectomy were collected at the Eye and ENT Hospital, Fudan University, China. The study was approved by the Institutional Review Board. All participants signed informed consent. Clinical information of the patients is provided in Table 1.

**Table 1.** Clinical characteristics of the patients enrolled in this study

Variables	Total patients (%)
**Age, years**	
<60	8 (26.7 %)
≥60	22 (73.3 %)
**Sex**	
Male	30 (100 %)
**Smoking history**	
Yes	30 (100 %)
**Drinking history**	
Yes	17 (56.7 %)
No	13 (43.3 %)
T staging*	
T1	2 (6.7 %)
T2	9 (30.0 %)
T3	12 (40.0 %)
T4	7 (23.3 %)
N staging*	
N0	23 (76.7 %)
N1	3 (10.0 %)
N2	4 (13.3 %）
TNM staging*	
Stage I–II	8 (26.7 %)
Stage III–IV	22 (73.3 %)

*TNM staging was based on the eighth edition of the American Joint Committee on Cancer Staging Manual.

### Quantitative real-time PCR (RT-qPCR)

Total RNA (1 µg) from fresh tumour tissues or the AMC-HN-8 cell line was converted to cDNA with an Evo M-MLV RT Mix Kit with gDNA Clean for qPCR (Accurate Biotechnology). RT-qPCR (10 µg reaction system) was then performed using an SYBR Green Premix Pro Taq HS qPCR Kit (Rox Plus) (Accurate Biotechnology) and ABI 7500 Real-Time PCR System (Thermo Fisher). We used the 2^−ΔΔCt^ method and calculated cycle threshold values to relative gene expression values. Glyceraldehyde 3-phosphate dehydrogenase (GAPDH) was set as an internal reference. The following primer sets were used: JAK3-F, 5ʹ-CCATCACGTTAGACTTTGCCA-3ʹ, JAK3-R, 5ʹ-GGCGGAGAATATAGGTGCCTG-3ʹ; and GAPDH-F, 5ʹ-TGTAGTTGAGGTCAATGAAGGG-3ʹ, GAPDH-R, 5ʹ-ACATCGCTCAGACACCATG-3ʹ. Three independent biological replicates and technical replicates were performed in each group. The reactions are given in Table 2 and the cycling conditions are given in Table 3.

**Table 2.** RT-qPCR system

Reagent	Volume (µl)
2X SYBR Green Pro Taq HS Premix (ROX plus)	5
Template	1
Primer F	0.2
Primer R	0.2
RNase-free water	3.6
Total	10

**Table 3.** RT-qPCR cycling conditions

	Temperature (°C)	Time (s)	Cycles
Step 1	95	30	1
Step 2	9560	530	40

### *F. nucleatum* quantification

Genomic DNA (gDNA) from fresh tumour tissues was extracted using the QIAamp BiOstic Bacteremia DNA Kit (Qiagen). *F. nucleatum* was detected by RT-qPCR (10 µg reaction system). We used the 2^−ΔCt^ method to calculate relative abundance. The reference gene was prostaglandin transporter (PGT). The following primer sets were used: *Fusobacterium nucleatum*-F, 5ʹ-CAACCATTACTTTAACTCTACCATGTTCA-3ʹ, *Fusobacterium nucleatum-*R*,* 5ʹ-GTTGACTTTACAGAAGGAGATTATGTAAAAATC-3ʹ; and prostaglandin transporter-F, 5ʹ-ATCCCCAAAGCACCTGGTTT-3ʹ, prostaglandin transporter-R, 5ʹ-AGAGGCCAAGATAGTCCTGGTAA-3ʹ.

## Results

### *F. nucleatum*-mediated DNA methylation in LSCC

**Fig. 1.**
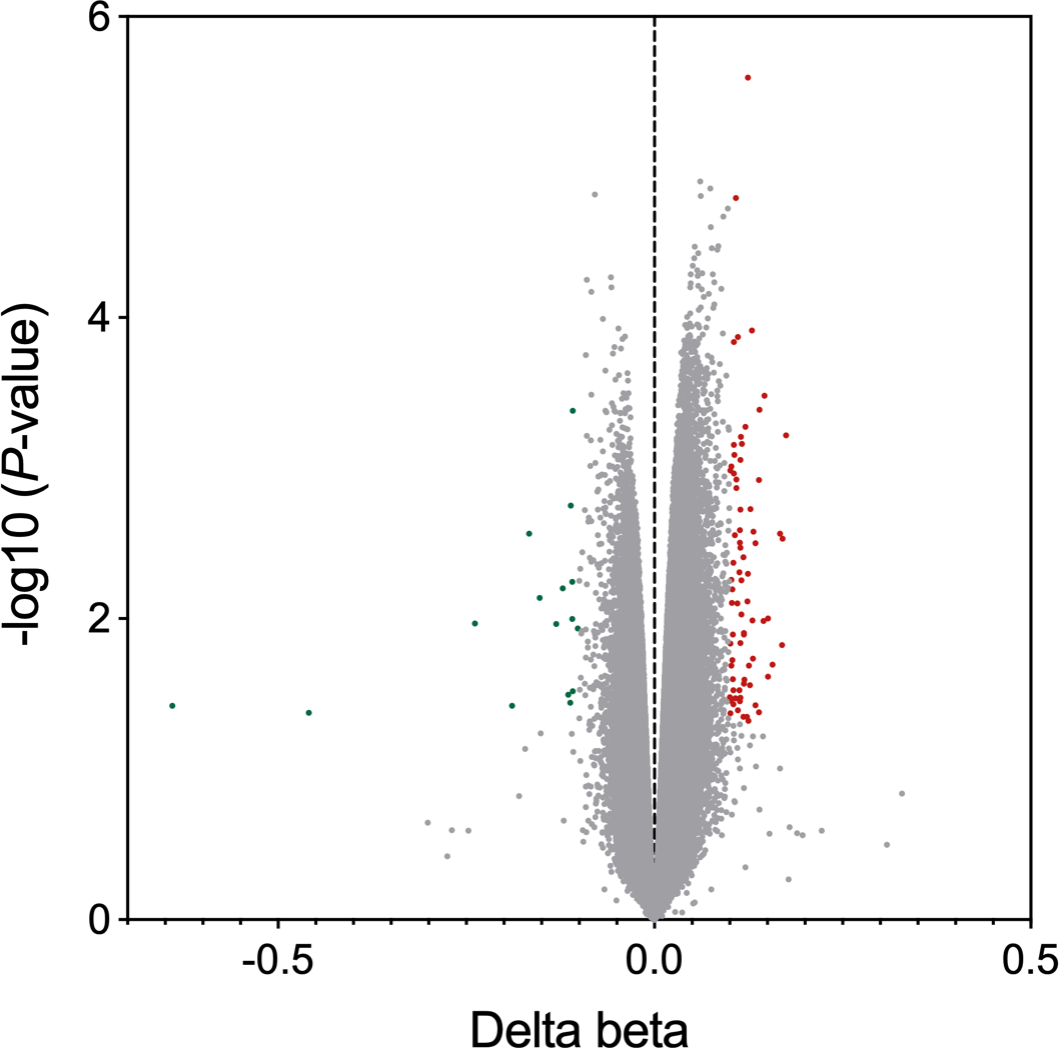
Volcano plot of the differential methylation sites. Red indicates hypermethylated sites and green indicates hypomethylated sites.

**Fig. 2.**
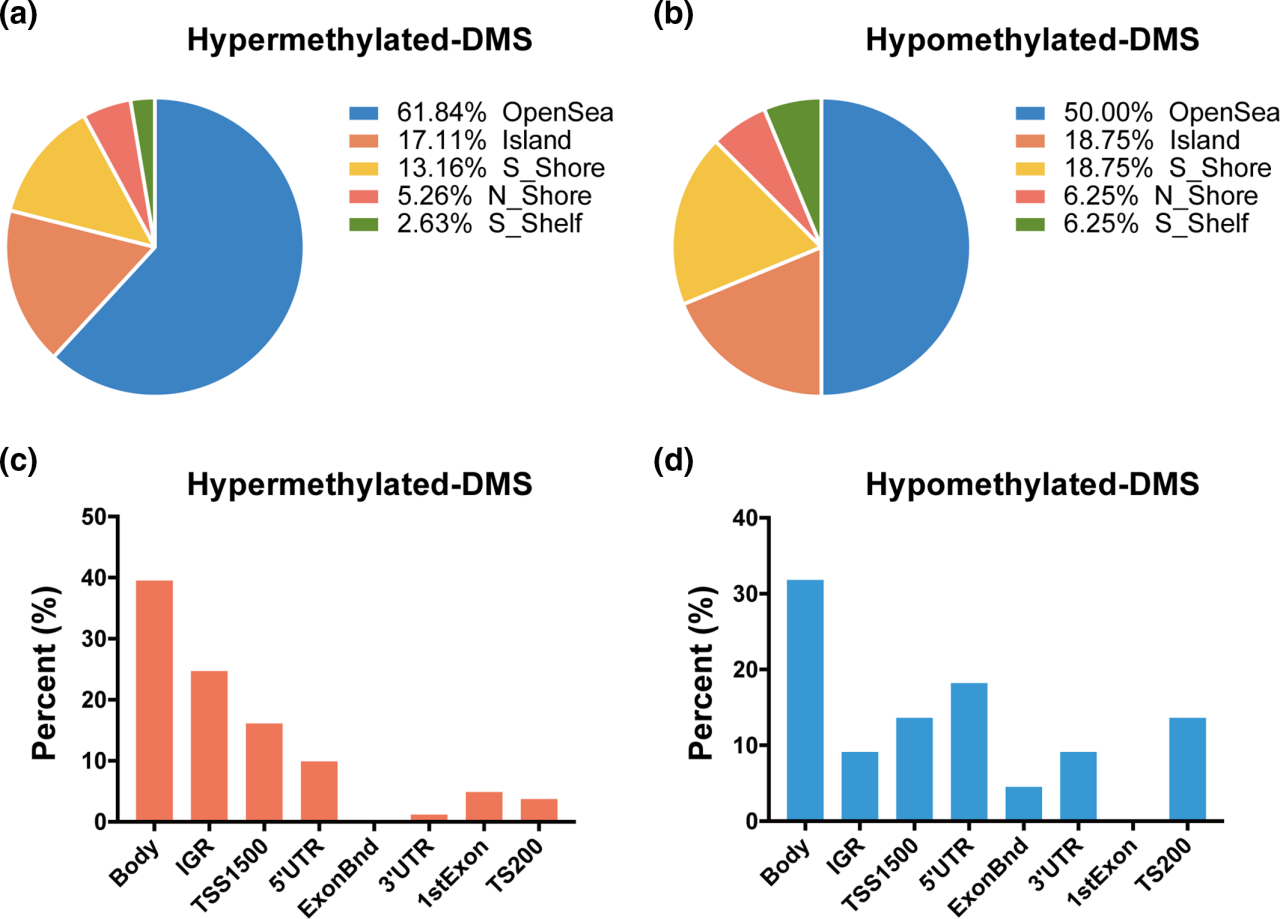
Distribution of the differential methylation sites (DMSs) across different genomic regions. (a, b) The proportions of hypermethylated and hypomethylated DMSs vary across different CpG island-related regions. (c, d) The proportions of hypermethylated and hypomethylated DMSs vary across different gene regions. IGR, intergenic region; TSS, transcription start site; UTR, untranslational region; 1stExon, first exon; ExonBnd, exon boundaries.

To examine whether *F. nucleatum* treatment affects the DNA methylation status in laryngeal cancer, a genome-wide methylation array was profiled using the 850K Illumina array after AMC-HN-8 cells were co-cultured with *F. nucleatum* for 24 h. A total of 92 significant DMSs were detected between the two groups (Fig. 1), of which 76 sites were hypermethylated and 16 were hypomethylated. CpG island annotation analysis revealed that 17.1 % of the hypermethylated DMSs were in CpG islands; 61.8 %, open sea; 13.2 %, S shore; 5.3 %, N shore; and 2.6 %, S shelf. The hypomethylation pattern of DMSs was similar to that of the hypermethylated DMSs, corresponding to: 18.8 %, CpG islands; 50.0 %, open sea; 18.8 % S shore; 6.3 %, N shore; and 6.3 %, S shelf in the aforementioned five regions (Fig. 2a, b). Further annotation according to gene locations showed that 34.6 % of hypermethylated and 45.4 % of hypomethylated DMSs were located in the promoter region (TSS200, TSS1500, 5′UTR and first exon), whereas 39.5 % of hypermethylated and 31.8 % of hypomethylated DMSs were located in the gene body (Fig. 2c, d). The annotation through human Genome Browser (University of California, Santa Cruz, UCSC, http://genome.ucsc.edu/) revealed that 92 DMSs were mapped to 76 genes, of which 57 were hypermethylated and 19 were hypomethylated.

### *F. nucleatum*-mediated gene expression in LSCC

**Fig. 3.**
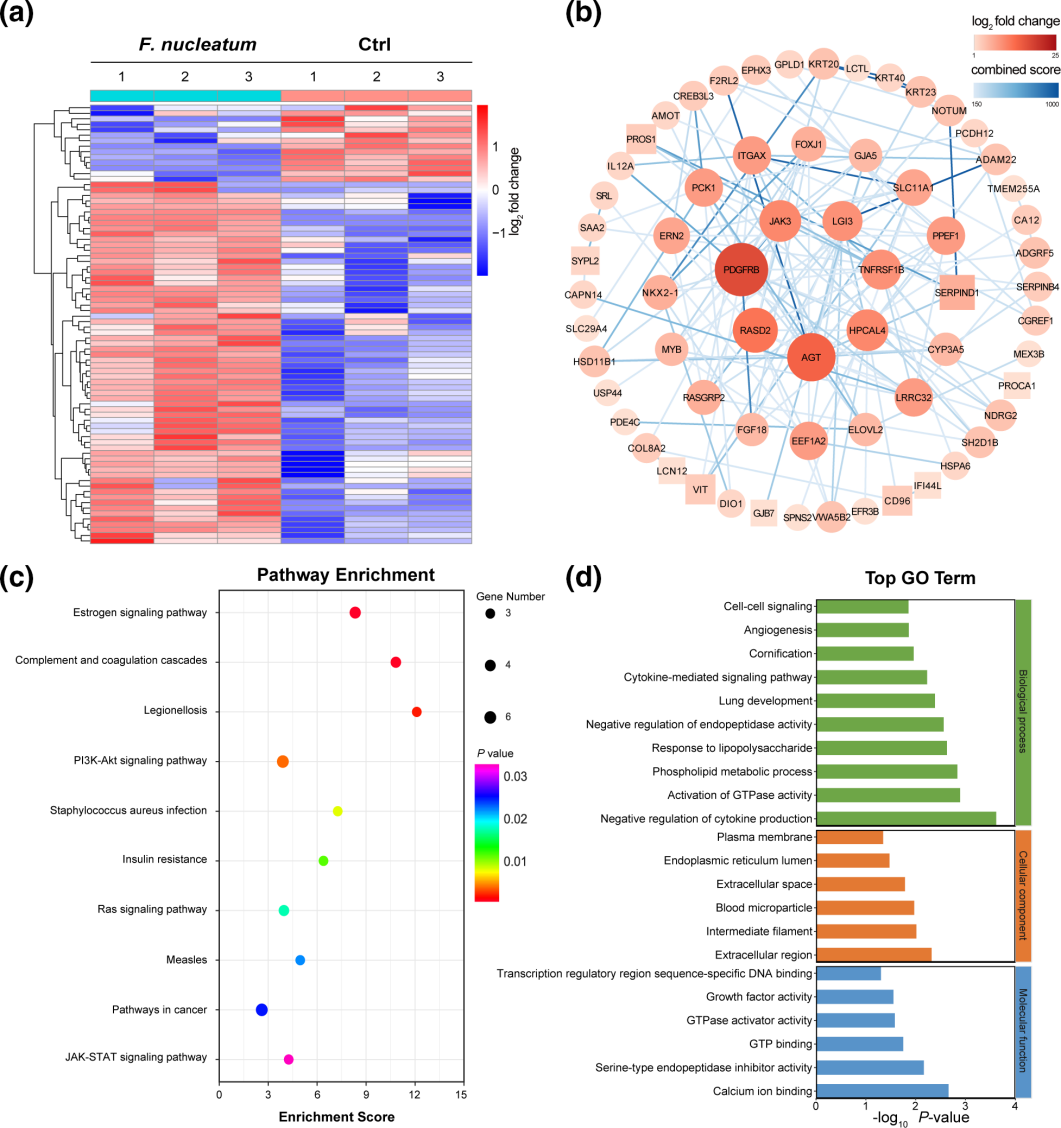
*F. nucleatum* regulates gene expression in laryngeal cancer. (**a**) Heatmap representing the differential gene expression patterns between *F. nucleatum* and control (Ctrl) groups (*P*<0.05, |log_2_ fold change|>1). (**b**) Protein–protein interaction network of the differentially expressed genes. Elliptical nodes represent upregulated mRNAs and rectangles represent downregulated mRNAs. Node colour shows the number of correlating nodes. Line colour shows the combined score. Node size is proportional to the number of connected nodes (degree). (**c**) The top 10 enriched Kyoto Encyclopedia of Genes and Genomes (KEGG) pathways of differentially expressed genes. (**d**) The top enriched Gene Ontology (GO) terms of differentially expressed genes.

To detect the effect of *F. nucleatum* on gene expression, transcriptome analysis was performed and the gene expression profiles were analysed. There were 66 upregulated genes and 14 downregulated genes in the *F. nucleatum* group (fold change >2, *P*<0.05) (Fig. 3a). The interaction network of the DEGs was constructed to further understand the interaction relationship (Fig. 3b). There were 65 nodes and 142 edges in the networks. The top seven DEGs with the highest degree of connectivity were platelet-derived growth factor receptor beta (PDGFRB), angiotensinogen (AGT), RASD family member 2 (RASD2), JAK3, hippocalcin-like protein 4 (HPCAL4), TNF receptor superfamily member 1B (TNFRSF1B) and leucine-rich repeat LGI family member 3 (LGI3). Meanwhile, analyses of KEGG pathway and GO term enrichment were conducted to ascertain the functional and biological roles of the DEGs in the *F. nucleatum* group. *F. nucleatum*-induced DEGs were notably involved in the oestrogen, phosphatidylinositol 3-kinase/protein kinase B (PI3K-Akt), tumorigenesis, and Janus kinase-signal transducer and activator of transcription (JAK-STAT) signalling pathways (Fig. 3c). The top enriched GO terms of the DEGs were those associated with the negative regulation of cytokine production in biological process (BP), extracellular region in cellular component (CC) and calcium ion binding in molecular function (MF) (Fig. 3d).

### Combined analysis of methylome and transcriptome data

**Fig. 4.**
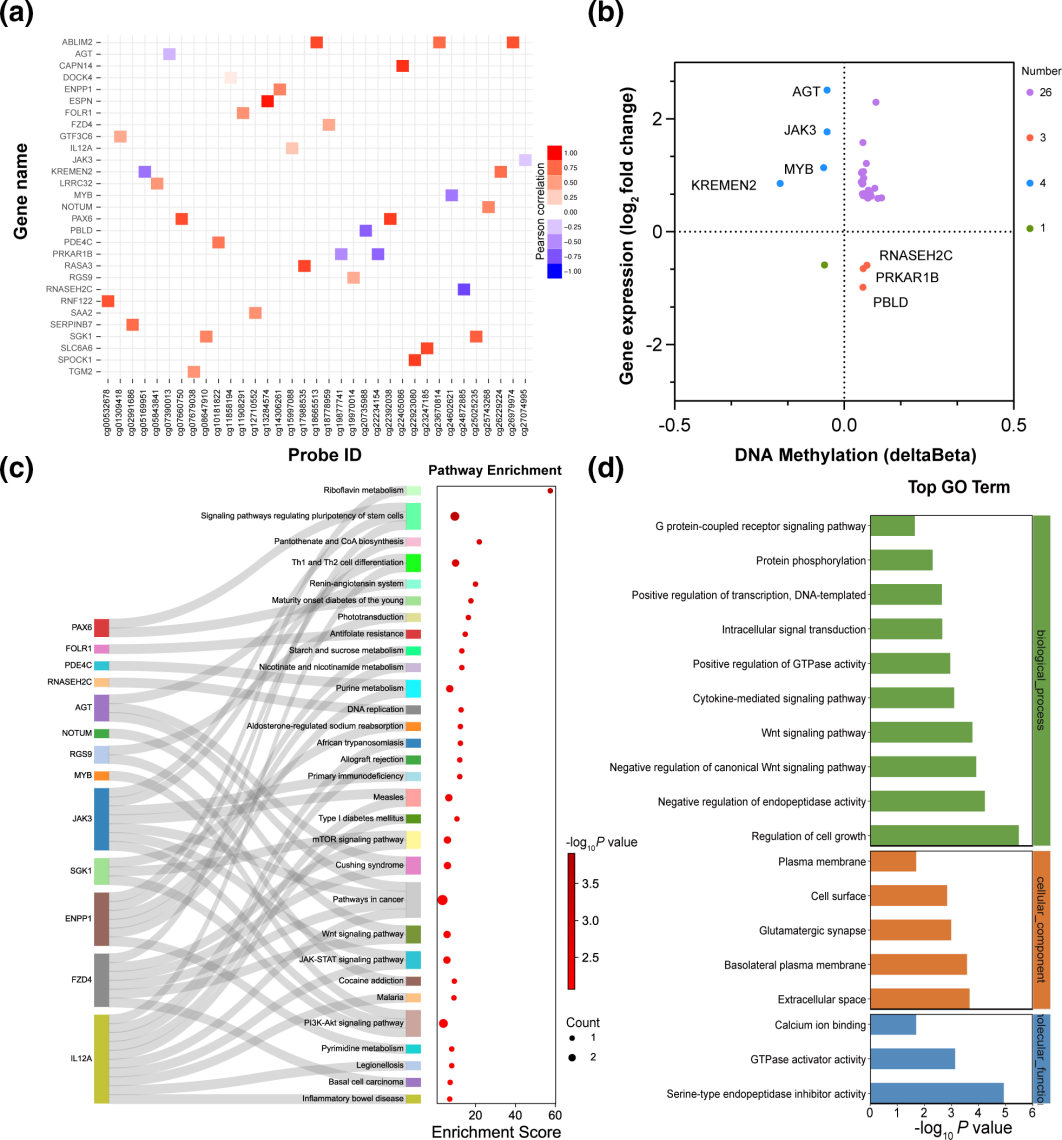
Combined analysis of methylome and transcriptome data. Heatmap (**a**) and four-quadrant plot (**b**) showing an association between differentially expressed genes and differential methylation sites. The genes were screened out with the threshold of *P*<0.05, |delta beta|>0.05 and fold change >1.5 or <0.5. (**c**) Sankey diagram visualizing the correlation of genes and KEGG pathways. (**d**) The top 30 enriched Gene Ontology terms.

We then conducted combined analysis of DMSs and DEGs (with the thresholds of |delta beta|>0.05 and fold change >1.5 or <0.5) to evaluate how DNA methylation affects gene expression. By mapping the methylation levels of significant DMSs to the expression levels of corresponding genes, 29 differentially expressed MDGs were identified (Fig. 4a). The four-quadrant diagram shows that four hypomethylated and 26 hypermethylated sites were associated with high mRNA expression, whereas one hypomethylated and three hypermethylated sites were associated with low mRNA expression (Fig. 4b). The expression of seven genes was negatively related to methylation levels; these included kringle-containing transmembrane protein 2 (KREMEN2) and ribonuclease H2 subunit C (RNASEH2C) located in the promoter regions, and AGT, MYB proto-oncogene transcription factor (MYB), JAK3, protein kinase cAMP-dependent type I regulatory subunit beta (PRKAR1B) and phenazine biosynthesis-like protein domain-containing (PBLD) located in the gene body. KEGG pathway and GO term enrichment analyses were performed for the 34 MDGs induced by *F. nucleatum* to investigate their functional significance. KEGG pathway analysis revealed that signalling pathways regulating riboflavin metabolism, the pluripotency of stem cells, and pantothenate and CoA biosynthesis were most significantly enriched. The proteins IL-12A and JAK3 were involved in most pathways. The pathways with the largest number of MDGs were those involved in tumorigenesis, signalling pathways regulating the pluripotency of stem cells, and the phosphatidylinositol 3-kinase/protein kinase B (PI3K-Akt) signalling pathway (Fig. 4c). Biological process, cellular component and molecular function were analysed based on GO analysis. The results showed that these MDGs were mostly enriched in the regulation of cell growth, extracellular space and serine-type endopeptidase inhibitor activity (Fig. 4d).

### *F. nucleatum* promotes JAK3 expression in LSCC

**Fig. 5.**
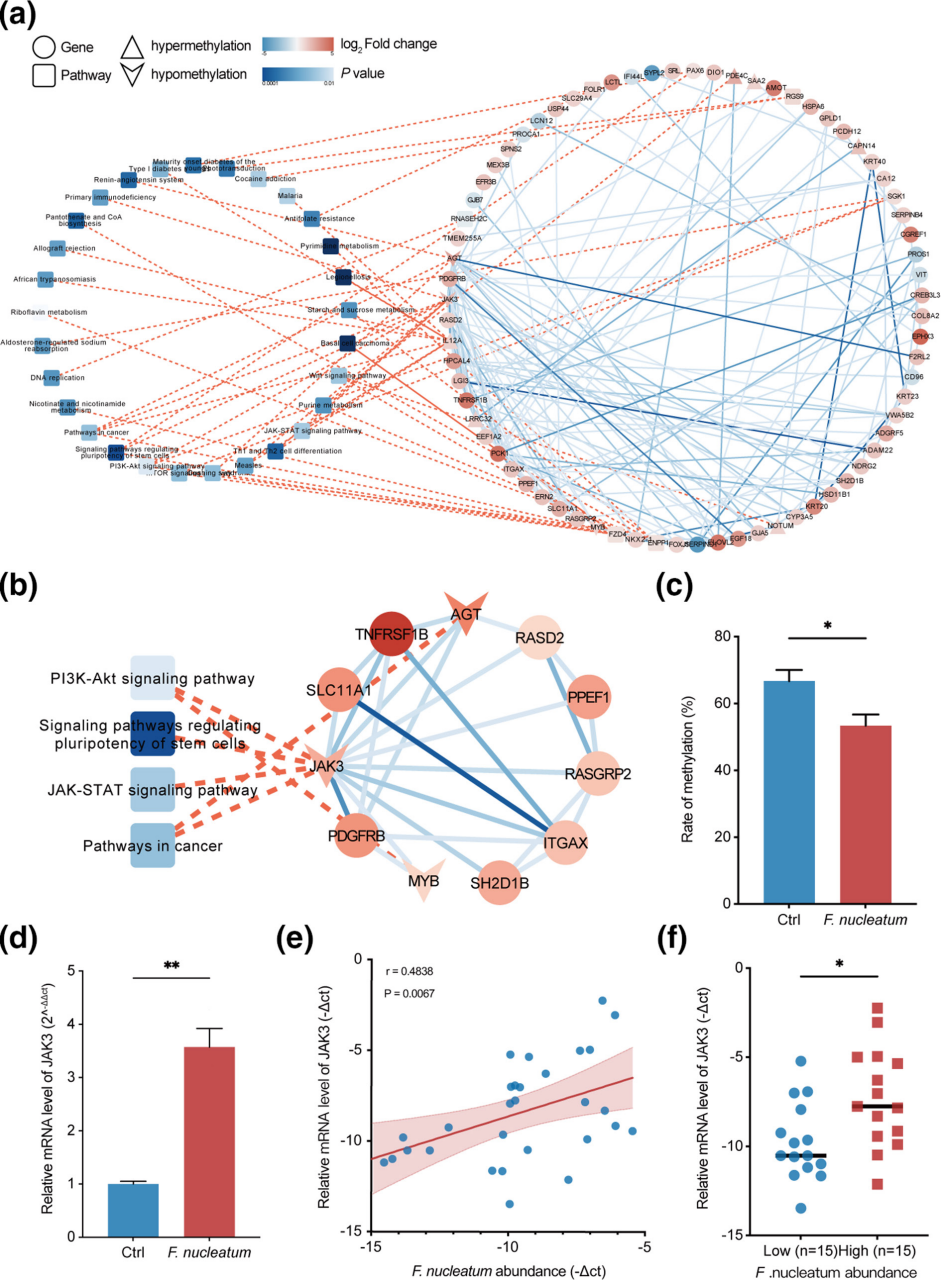
*F. nucleatum* promotes the expression of Janus kinase 3 (JAK3) in laryngeal squamous cell carcinoma (LSCC). (**a**) Visualization of the protein–protein interaction network combining methylation and KEGG pathway data. (**b**) JAK3 network combining methylation and KEGG pathway data. (**c**) JAK3 DNA methylation levels in *F. nucleatum*-treated LSCC cells (*n*=3 per group). (**d**) JAK3 gene expression in *F. nucleatum*-treated LSCC cells (*n*=3 per group, mean±sem). (**e**) Correlation between *F. nucleatum* abundance and relative mRNA levels of JAK3 in LSCC tissues (*n*=30). (**f**) Relative mRNA levels of JAK3 at a high and low abundance of *F. nucleatum*. *P ＜ 0.05, **P ＜ 0.01.

After combining the DNA methylation and KEGG pathway data with the interaction network data (Fig. 5a), we found that JAK3 was involved in several signalling pathways relating to cancer, including the JAK-STAT, tumorigenesis and PI3K-Akt signalling pathways, suggesting that *F. nucleatum* may regulate cancer development and metastasis through JAK3 (Fig. 5b). The methylation and expression levels of JAK3 were measured to validate the results obtained through the 850K BeadChip arrays and transcriptome sequencing data. BSP analysis was used to determine the methylation status of JAK3 in *F. nucleatum*-treated LSCC cells. The results demonstrated that treatment with *F. nucleatum* reduced the DNA methylation of JAK3 in comparison to the control group (Fig. 5c). RT-qPCR analysis revealed that the *F. nucleatum-*treated group showed a significant upregulation of JAK3 levels (Fig. 5d). For further validation, we investigated the tumour tissues from 30 patients with laryngeal carcinoma. Notably, *F. nucleatum* abundance in tumour tissues correlated positively with JAK3 expression (Fig. 5e). After dividing patients into two groups according to the median relative abundance of *F. nucleatum*, we observed that JAK3 expression showed a significant increase in the high *F. nucleatum* abundance group (Fig. 5f).

## Discussion

DNA methylation is considered a key regulater in epigenetic process wherein, most commonly, a methyl group is transfered to the cytosine′ C5 position, termed 5-methyl-cytosine (5-mC), and regulates gene expression. In this study, the methylation sites of *F. nucleatum*-treated laryngeal cancer cells were evaluated to determine *F. nucleatum*-mediated epigenetic methylation-related mechanisms. In total, we identified 92 DMSs, most of which were located in the gene body and open sea region relative to CpG islands. Further combined analysis of methylome and transcriptome data revealed 29 differentially expressed MDGs.

The significance of this study lies in exploring the effect of *F. nucleatum* on DNA methylation. Accumulating evidence indicates the association between *F. nucleatum* and tumour metastasis. *Fusobacterium* species in metastatic tumours are highly similar to those found in primary tumours, suggesting that the microbiota maintains stability between primary and metastatic tumours [18]. Reportedly, *F. nucleatum* is significantly increased in patients with lymph node metastasis and *F. nucleatum* upregulates keratin 7-antisense/keratin 7 (KRT7-AS/KRT7) through the nuclear factor kappa B pathway and promoted CRC cell migration [19]. *F. nucleatum* regulates the TLR4/MYD88/miR-205–5 p pathway and induces epigenetic alterations, thus promoting DNA damage and cell proliferation [19]. *F. nucleatum* induces the epithelial–mesenchymal transition (EMT) by regulating the long non-coding RNA MIR4435-2HG [20]. Our previous study revealed that *F. nucleatum* promoted LSCC progression and metastasis by suppressing transforming growth factor, beta receptor II (TGFβR2) expression, resulting in activation of the PI3K-Akt pathway and attenuation of the TGF-β/Smad pathway. Interestingly, restoring TGFβR2 expression resulted in an unexplained multisite metastasis, demonstrating that *F. nucleatum* facilitates tumour metastasis through other pathways. An in-depth investigation of *F. nucleatum* in metastasis may help to ameliorate the prognosis of LSCC.

The current study further revealed that *F. nucleatum* may upregulate the expression of JAK3 through gene methylation, thereby regulating the JAK-STAT and PI3K-Akt signalling pathways. Since the PI3K-Akt pathway plays an important role in the control of cell proliferation and gene expression and protein synthesis, it is crucial for carcinogenesis. Moreover, the PI3K-Akt pathway is a key characteristic of EMT [21]. The pro-tumorigenic effect of bacteria via the PI3K-Akt pathway in CRC has been reported [22]. The PI3K-Akt pathway has also been linked to metastasis in head and neck cancers [23,25]. Our previous study revealed that *F. nucleatum* activated the PI3K-Akt pathway and promoted EMT by inhibiting TGFβR2 in laryngeal cancer [26]. Meanwhile, the JAK-STAT pathway plays a major role in *F. nucleatum*-induced tumour metastasis and is known to participate in the tumorigenesis and metastasis of human cancer. Stephen *et al*. reported that the erythropoietin-activated JAK-STAT signalling pathway [27] promoted tumour invasion in head and neck cancers. In oral cancer, expression of cell motility and melatonin-regulated oral cancer stimulator-1/protein expression of prune homolog 2 (MROS-1/PRUNE2) were significantly controlled by melatonin via activation of the JAK-STAT pathway [28]. By modifying the JAK-STAT pathway in carcinogenesis, the vitamin D receptor protects mice from dysbiosis [29], which is critical for maintaining microbial and intestinal homeostasis.

In this study, we demonstrated that *F. nucleatum* treatment led to altered DNA methylation patterns in laryngeal cancer, and hence affected gene expression. Epidemiological studies have demonstrated that increased levels of *F. nucleatum* are associated with microsatellite instability, the CpG island methylator phenotype and genomic mutations [4]. *F. nucleatum* was confirmed to cause promoter hypermethylation of tumour suppressor genes by increasing the expression of DNMT1 and DNMT3A (DNA methyltransferase) *in vitro* and *in vivo* [30]. However, the expression of a majority of genes was negatively related to methylation at the promoter region and positively related to methylation in the gene body. We observed a negative correlation between gene methylation and expression in the body region, but a positive correlation in the promotor region. A few possible explanations could account for these findings. First, gene body methylation was complexly related to gene expression, and in some cases, the relationship is merely correlative rather than causative [31,32]. We observed an alternation in both gene expression and gene body methylation. Second, gene body methylation is only one of the many factors that influence gene expression, which may also be regulated via histone modification [33]. Additionally, DNA methylation in microRNA (miRNA) promoters can also control miRNA transcription levels, thereby altering target gene expression [34].

We need to consider certain limitations of this study. We have observed that *F. nucleatum* promoted JAK3 gene expression. The combined methylome and transcriptome analysis, however, did not indicate the direct mechanism that *F. nucleatum* exerts on epigenetic events. A deeper understanding of the specific mechanisms behind the promotion remains to be done. Also, our work cannot demonstrate the direct effect of methylation in transcriptional regulation. This study was conducted on only one cell line, and it is important to note that our sample size was relatively small. A larger sample size for the detection of *F. nucleatum* abundance and JAK3 expression is needed to verify our findings.

In conclusion, our study is the first study to examine the *F. nucleatum*-mediated methylation profile of laryngeal cancer. We identified the whole genome-wide methylation map and screened DEGs. We observed that *F. nucleatum* treatment induced demethylation and upregulation of JAK3, contributing to activation of the PI3K-Akt and JAK-STAT signalling pathway, and potentially participating in tumour metastasis. This analysis provides a better understanding of the role that *F. nucleatum* plays as a epigenetic regulator in the metastasis of LSCC. The identification of these methylation patterns offers new possibilities for mechanistic studies to explore therapeutic strategies in laryngeal cancer.
